# Reply: Early onset of breast cancer in black British women: how reliable are the findings?

**DOI:** 10.1038/sj.bjc.6604626

**Published:** 2008-08-26

**Authors:** S W Duffy, R L Bowen, D A Ryan, I R Hart, J L Jones

**Affiliations:** 1 Institute of Cancer and Cancer Research UK Clinical Centre, Centre for Tumour Biology Barts and the London Queen Mary's School of Medicine and Dentistry John Vane Science Centre, Charterhouse Square, London EC1M 6BQ, UK

**Sir,**

It is reassuring that Dr Ingleby reaches the same conclusion as we did, that black patients have earlier onset on average than white patients, even after taking into account the different age distributions of the populations. Unlike Dr Ingleby, we are reluctant to attempt to deduce absolute incidence from our figures, as hospital admissions do not exactly correspond to geographical areas. Therefore, we cannot be certain that the differences are entirely because of the ‘black other’ subgroup, as suggested by Dr Ingleby. Although the ‘black other’ category has the youngest age at onset in our data, the black African, Caribbean and British patients were also significantly younger than white patients (*P*<0.001). The poorer survival in tumours 2 cm in size or less was also observed in all the black ethnic subgroups.

Dr Ingleby queries the effect of socioeconomic status. As measured by the Index of Multiple Deprivation, this did not differ significantly between ethnic groups, nor did it have a significant effect on survival.

We have taken care to avoid knee-jerk reactions, such as to recommend an early detection response to these observations. The fact that the poorer survival of black patients was confined to small tumours suggests that early detection approaches are not the solution, or at least not the whole solution. Our next task is the further study of aetiology and tumour biology to find out why there is earlier onset among black patients and why they have poorer survival in the case of small tumours. When these questions have been answered, we will be in a better position to prescribe appropriate control measures.

